# Preventive Effect of Hippocampal Sparing on Cognitive Dysfunction of Patients Undergoing Whole-Brain Radiotherapy and Imaging Assessment of Hippocampal Volume Changes

**DOI:** 10.1155/2022/4267673

**Published:** 2022-04-05

**Authors:** Weijie Shang, Hongmin Yao, Ying Sun, Anna Mu, Li Zhu, Xia Li

**Affiliations:** ^1^Oncology Department, Shenyang Tenth People's Hospital, Shenyang Chest Hospital, China; ^2^Radiation Oncology Department of Head and Neck Cancer, Cancer Hospital of China Medical University, Liaoning Cancer Hospital & Institution, China

## Abstract

**Objective:**

Preventive effect of hippocampal sparing on cognitive dysfunction of patients undergoing whole-brain radiotherapy and imaging assessment of hippocampal volume changes.

**Methods:**

Forty patients with brain metastases who attended Liaoning Cancer Hospital from January 2018 to December 2019 were identified as research subjects and were randomly divided into a control group and an experimental group, with 20 cases in each group. The control group was treated with whole-brain radiotherapy (WBRT), and the experimental group was treated with hippocampal sparing-WBRT (HS-WBRT). The Montreal Cognitive Assessment (MoCA) score, Eastern Cooperative Oncology Group (ECOG) score, cancer quality-of-life questionnaire (QLQ-C3O) score, hippocampal volume changes, and prognosis of the two groups were compared.

**Results:**

The MoCA scores decreased in both groups at 3, 6, and 12 months after radiotherapy, with significantly higher scores in the experimental group than in the control group (*P* < 0.05). After radiotherapy, both groups had lower ECOG scores, with those in the experimental group being significantly lower than those in the control group (*P* < 0.05). After radiotherapy, the QLQ-C30 score was elevated in both groups, and that of the experimental group was significantly higher than that of the control group (*P* < 0.05). The experimental group outperformed the control group in terms of the prognosis (*P* < 0.05). The hippocampal volume of the control group was significantly smaller than that of the experimental group (*P* < 0.05).

**Conclusion:**

The application of hippocampal sparing in patients receiving whole-brain radiotherapy is effective in preventing cognitive dysfunction, improving the quality of life and prognosis of patients, and avoiding shrinkage of hippocampal volume.

## 1. Introduction

Brain metastases mostly indicate an advanced stage of tumors, which may seriously threaten the life safety of patients in case of delayed treatment [1]. Clinically, the treatment of brain metastases is primarily aimed at controlling tumor growth, reducing patients' pain, prolonging their survival time, and improving their cognitive function and quality of life [2, 3]. Conventional whole-brain radiotherapy (WBRT) is the current standard radiotherapy regimen for brain metastases and preventive brain irradiation, including WBRT and stereotactic radiotherapy, which may also damage nerve cells, leading to cognitive dysfunction and compromised quality of life [4–6]. The hippocampus is an important tissue in the brain for information and memory storage. Neural stem cells in the hippocampus are strongly sensitive to radiation, and the dose of irradiation to the hippocampus during radiotherapy directly affects the rate of proliferation and apoptosis of neural stem cells. Over 90% of brain metastases occur 5 mm outside the hippocampus, so the side effects of WBRT during irradiation can be attenuated by hippocampal sparing [7–9]. Hippocampal sparing is a major difficulty in WBRT, in which the key is to limit hippocampal exposure during radiotherapy to reduce cognitive impairment in patients [10–12]. Accordingly, this study was aimed at the preventive effect of hippocampal sparing on cognitive dysfunction of patients undergoing whole-brain radiotherapy and imaging assessment of hippocampal volume changes. The results are as follows.

## 2. Materials and Methods

### 2.1. Ethical Statement

The study was approved by the Hospital Ethics Committee, and the patients and their families were informed of the purpose and process of the experimental study and signed the informed consent.

#### 2.1.1. General Information

Forty patients with brain metastases who attended Liaoning Cancer Hospital from January 2018 to December 2019 were identified as research subjects and were randomly divided into a control group and an experimental group, with 20 cases in each group.

#### 2.1.2. Inclusion Criteria

The inclusion criteria were as follows: (1) patients with clear pathological diagnosis of malignant tumors; (2) patients with small cell lung cancer brain prophylactic cranial irradiation (PCI) or multiple brain metastases (≥4 brain metastases) from other malignant tumors; (3) patients with metastases at a distance of more than 2 cm from the hippocampus; (4) patients aged 18 years-80 years; (5) patients with a KPS score ≥ 70 and PS score ≤ 2 before radiotherapy; (6) patients without significant bone marrow suppression and hepatic and renal impairment; (7) patients without serious comorbidities (e.g., heart disease, tuberculosis); (8) the study was approved by the hospital ethics committee, and patients and their family members were informed of the purpose and procedure of the study and signed the informed consent form; (9) patients with an expected survival of >12 months.

#### 2.1.3. Exclusion Criteria

The exclusion criteria were as follows: (1) patients with severe systemic diseases such as hepatic or renal insufficiency and heart failure, (2) patients with other severe acute or chronic diseases, and (3) patients with severe psychiatric diseases that affect the progress of the study

### 2.2. Methods

After enrollment, all patients underwent brain imaging, routine blood tests, liver and kidney function tests, and electrocardiogram (ECG) inspection. The apparatus and equipment used in the study were presented as follows: 3.0 T MRI: Siemens, Germany; ECG machine: Shanghai Photoelectric; ARCJOTECTi2000 automatic chemiluminescence immunoassay analyzer: USA; XN-10 [B4] automatic modular blood and body fluid analyzer: Japan; thermostatic sink: Guangzhou Clinoradi Company; thermoplastic mask: Guangzhou Clinoradi Company; SOMATOM Definition AS Large aperture spiral positioning CT: Siemens, Germany; Pinnacle 9.10 planning system: Philips, USA; Tomotherapy HD: Accuray, USA; IX SN6117, treatment bed, and headboard: Varian, USA. The specific methods of irradiation techniques for both groups are shown in Table 1.

*Computed tomography (CT) simulation positioning*: in both groups, simulation positioning was performed on the patient under a large-aperture spiral CT flat-scan. Patients were in supine position on a positioning bed with postural fixation devices, and their positions were secured with headrests and thermoplastic masks. The scan area was the whole brain (upper border to the cranial vault and lower border to the C2 level) with a layer thickness of 2 mm, and the images were transferred to the Pinnacle 9.10 treatment planning system (TPS).

*Magnetic resonance imaging (MRI) positioning*: a brain contrast-enhanced 3D MRI was performed, and T1- and T2-weighted sequences with T1-weighted enhanced sequences were acquired with a layer thickness of 2 mm. The CT and MRI images (cranially based) were merged for image alignment on the Varian Pinnacle 9.10, and the hippocampus was contoured on the T1-weighted images.

*CT-MRI fusion*: the hippocampal sparing group underwent brain contrast-enhanced MRI with a scan layer thickness requirement of 2 mm, consistent with CT simulation positioning. Image alignment fusion of CT images with MRI images on the Pinnacle 9.10 was performed by the physiatrist, and the hippocampal region was contoured on the merged images.

**Figure 1:**
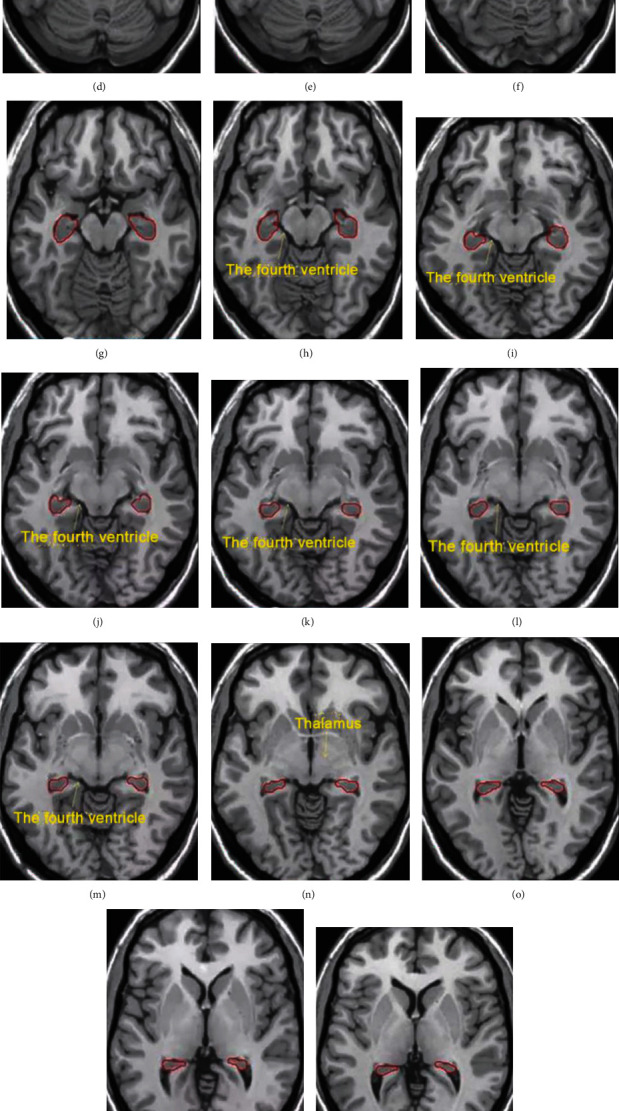
Hippocampal contouring atlas. Note: (a) beginning level (beginning of the most inferior pole of the hippocampus). The gray matter areas medial to the lateral ventricles were delineated starting at the emergence of the inferior horn of the lateral ventricle, and the white matter areas were not delineated. (b) The delineation of the gray matter area in the inferior horn of the lateral ventricle. (c) The posterior hyphae (white matter area) was omitted. (d) As the level moved up, the hippocampus gradually moved posteriorly, omitting the posteriorly located germinal hairs and the anteriorly located amygdala. (e) The anteriorly located amygdala and hook-shaped echo. (f) The uncal recess in the inferior horn of the lateral ventricle appeared, behind which was the hippocampus. The medial margin of the hippocampus does not exceed the vertical line of the uncal recess. (g) The delineation was continued upward. The gray matter area anterior to the germinal hairs and the inferior horn of the lateral ventricle was omitted from delineation. (h) The fourth ventricle appeared, and the medial border of the hippocampus was bordered by the fourth ventricle. (i) The inferior horn of the lateral ventricle started to become blurred, and the medial border of the hippocampus was bordered by the fourth ventricle. (j) The fourth ventricle as the boundary. (k) The fourth ventricle as the boundary. (l) The fourth ventricle as the boundary. (m) The fourth ventricle as the boundary. (n) The caudal part of the hippocampus was located posterior to the thalamus and curved toward the uncal recess. (o) The caudal part of the hippocampus extended posteriorly to the anteromedial side of the lateral ventricles. (p) The caudal part of the hippocampus extended posteriorly to the anteromedial side of the lateral ventricles. (q) The caudal part of the hippocampus remained at the lateral border of the fourth ventricle during the continuous upward scanning. (r) The display of the caudal part of the hippocampus terminated in the appearance of white matter between the lateral ventricles and the gray matter of the hippocampus.

*Hippocampus contouring*: the hippocampus was contoured in T1-weighted sequence axial slices, which was defined as the hippocampal region, and a hippocampal protected region (PRV) was then created, where the hippocampus expanded 5 mm outward in the three-dimensional direction, to allow for a dose drop between the hippocampus and the planned target area (PTV). First, the most clearly defined level of the lateral subventricular horn was identified on the transverse truncated T1-weighted MRI image, and the encapsulated gray matter area in the lateral subventricular horn was the hippocampus (i.e., the anterior, medial, and lateral borders of the hippocampus were the cerebrospinal fluid, and the posterior border was the white matter). Second, the hippocampus was continued to be delineated in the direction of the peduncle. The anterior border of the hippocampus was difficult to distinguish from the amygdala, which requires estimation based on the border of the upper layer, with the cerebrospinal fluid and white matter on the lateral sides and the posterior border and the pituitary level on the inferior border. Third, following the first step, the hippocampus was contoured in the cephalic direction in the region between the temporal lobe and the midbrain and thalamus. The medial and lateral borders were the cricoid pool and the lateral ventricles of the brain, respectively; the anterior border was the inferior horn of the lateral ventricles and the white matter; the posterior and superior borders were white matter and the superior lateral part of the ventricles, with the upper border adjacent to the level of the splenium of corpus callosum. The prescribed dose of PTV for WBRT was 30 Gy, 3 Gy/time, 5 times/week, and some patients were given shrinking field dimensions. For planned optimization goals, >95% of PTV volume received the prescribed dose, lens dose < 8 Gy, average eye dose < 20 Gy, and hippocampus 1% volume dose < 24 Gy. The hippocampal contouring atlas is shown in Figure 1.

**Figure 2:**
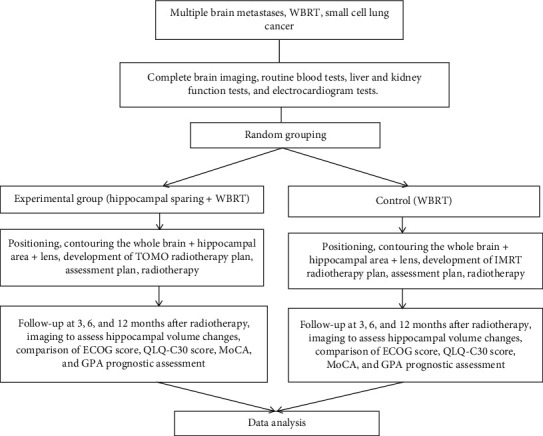
Study roadmap.

The examinations of the enrolled patients were completed in our hospital, and the detailed study roadmap is shown in Figure 2.

### 2.3. Observation Indicators and Evaluation Criteria

1. The assessment of cognitive function was completed using the Montreal Cognitive Assessment (MoCA), which includes 11 examination items in 8 cognitive domains, including attention and concentration, executive function, memory, language, visual-structural skills, abstract thinking, computation, and orientation, with a total score of 30 points and ≥26 points was considered a normal cognitive function. One point was added to the cognitive function score for years of education ≤ 12 years. The scale was completed before radiotherapy and 3, 6, and 12 months after the radiotherapy
2. ECOG scoring criteria. A score of 0 indicates that mobility is completely normal and does not differ from that before the onset of the disease. A score of 1 indicates the ability to walk freely and engage in light physical activities, including general housework or office work, but not in heavier physical activities. A score of 2 indicates that the patients can walk freely with self-care ability and can perform out-of-bed activities at least half of the daytime, but they have lost the ability to work. A score of 3 indicates partial self-care, being bedridden, or wheelchair-bound for more than half of the daytime. A score of 4 indicates bedridden and the loss of self-care ability. A score of 5 indicates death. The lower the score, the better the physical condition
3. The cancer quality-of-life questionnaire (QLQ-C3O) (V3.0) was used to evaluate patients' quality of life. There are five functional domains in the QLQ-C30 (V3.0), including somatic, role, cognitive, emotional, and social functioning. The higher the score, the better the quality of life
4. The prognostic impact of brain metastases on the prognosis of patients in both groups was assessed using the graded prognostic assessment (GPA) for patients with brain metastases. The system included four prognostic factors (age, KPS score, number of brain metastases, and extracranial metastases), and the total GPA score was the sum of the scores of the four prognostic factors, which were divided into four classes according to the GPA score. The specific scoring criteria and their relationship with the median survival of patients are shown in Tables 2 and 3

### 2.4. Statistical Methods

The data processing software selected for this study was SPSS 20.0, and GraphPad Prism 7 (GraphPad Software, San Diego, USA) was used to plot the graphics of the data. Count data were expressed by *n* (%) using the chi-square test, and measurement data were expressed by ($\;\overset{¯}{x}\pm s$) using the *t*-test. Differences were statistically significant when *P* < 0.05.

## 3. Results

### 3.1. Comparison of General Information

The general information of the two groups of patients was comparable (*P* > 0.05). The details are shown in Table 4.

### 3.2. Comparison of MoCA Scores

There was no statistically significant difference in MoCA scores between the two groups before radiotherapy (*P* > 0.05). The MoCA scores decreased in both groups at 3, 6, and 12 months, with higher MoCA scores observed in the experimental group than the control group (*P* < 0.05) (see Table 5).

### 3.3. Comparison of ECOG Scores

**Figure 3:**
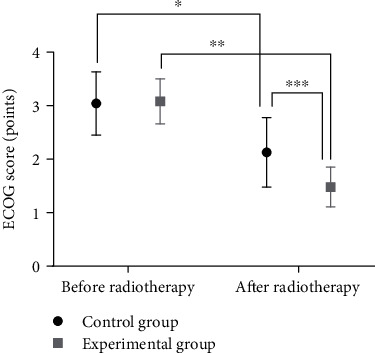
Comparison of ECOG scores before and after radiotherapy between the two groups ($\overset{¯}{x}\pm s$). Note: the abscissa represents the control group and experimental group, and the ordinate represents the ECOG score, points. The ECOG scores before and after radiotherapy in the control group were 3.04 ± 0.59 and 2.13 ± 0.65, respectively. The ECOG scores before and after radiotherapy in the experimental group were 3.08 ± 0.42 and 1.48 ± 0.37, respectively. ∗ indicates a significant difference in the ECOG scores before and after radiotherapy in the control group (*t* = 4.636, *P* < 0.001). ∗∗ indicates a significant difference in the ECOG scores before and after radiotherapy in the experimental group (*t* = 12.784, *P* < 0.001). ∗∗∗ indicates a significant difference in the ECOG scores between the two groups after radiotherapy (*t* = 3.887, *P* < 0.001).

There was no statistically significant difference in ECOG scores between the two groups before radiotherapy (*P* > 0.05). After radiotherapy, the ECOG scores improved in both groups, in which the experimental group had lower ECOG scores than the control group (*P* < 0.05), as shown in Figure 3.

### 3.4. Comparison of QLQ-C30 Scores

**Figure 4:**
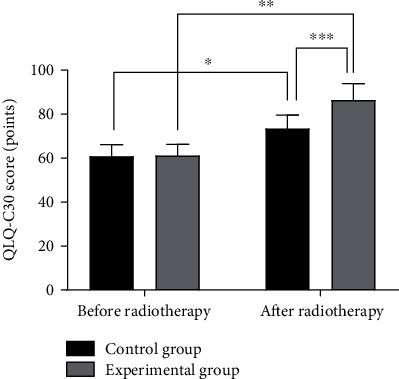
Comparison of QLQ-C30 scores before and after radiotherapy between the two groups ($\overset{¯}{x}\pm s$). Note: the abscissa represents the control group and the experimental group, and the ordinate represents the QLQ-C30 score, points. The QLQ-C30 scores before and after radiotherapy in the control group were 60.75 ± 5.34 and 73.48 ± 6.02, respectively. The QLQ-C30 scores before and after radiotherapy in the experimental group were 61.19 ± 5.12and 86.42 ± 7.55, respectively. ∗ indicates a significant difference in the QLQ-C30 scores before and after radiotherapy in the control group (*t* = 7.075, *P* < 0.001). ∗∗ indicates a significant difference in the QLQ-C30 scores before and after radiotherapy in the experimental group (*t* = 12.369, *P* < 0.001). ∗∗∗ indicates a significant difference in the QLQ-C30 scores between the two groups after radiotherapy (*t* = 5.993, *P* < 0.001).

The two groups showed no statistically significant difference in QLQ-C30 scores before radiotherapy (*P* > 0.05). After radiotherapy, the QLQ-C30 scores of both groups were improved, with higher results in the experimental group than the control group (*P* < 0.05) (see Figure 4).

### 3.5. Comparison of Clinical Prognosis

The experimental group outperformed the control group in terms of the prognosis of patients (*P* < 0.05), as detailed in Table 6.

## 4. Discussion

Whole-brain radiation therapy is an essential method for treating brain metastases, which is widely used in clinical practice. Irradiation of the target area may trigger radiation damage to the normal brain tissue adjacent to the tumor, resulting in demyelination changes in the white matter of the brain, cerebral edema, and damage to the vascular endothelium, which may significantly compromise the cognitive function of the patient. Therefore, hippocampal sparing is of great importance to patients undergoing WBRT [13, 14]. The hippocampus, located in the medial temporal lobe of the brain, is a vitally important structure in the limbic system of the brain, which is highly involved in learning, memory, emotion, motor control, and endocrine regulation and is also a reservoir for short-term memory [15, 16]. The hippocampus is composed of gray matter and white matter; the gray matter includes the hippocampus, dentate gyrus, hypothenar gyrus, fasciculus, and gray tegument, and the white matter part includes the hippocampal umbrella, dome foot, dome union, and dome body [17, 18]. Related studies have shown that hippocampal tissue is very sensitive to radiation, and low doses of radiation may result in damage to the hippocampal region, which will manifest as progressive irreversible memory loss and dementia, severely compromising the quality of life of patients [19, 20]. Hippocampal sparing whole-brain radiotherapy (HS-WBRT), which limits the maximum line volume of the hippocampus to less than 10 Gy and ensures the accuracy of the hippocampal region outline, is an intensity-modulated radiotherapy technique that requires a reasonable irradiation field according to the position of the hippocampus in the skull [21–23].

In this study, the two groups showed comparable MoCA scores before radiotherapy (*P* > 0.05), and the MoCA scores decreased at 3, 6, and 12 months after the therapy, with higher scores in the experimental group than the control group (*P* < 0.05). The results are consistent with the research results of Firth et al. [24], indicating that WBRT may impair the cognitive functions of patients, and the hippocampus sparing alleviates the impairment of the cognitive functions. Moreover, the ECOG scores, which were similar in the two groups before radiotherapy, were reduced significantly after the therapy, with lower results in the experimental group than the control group. The posttherapeutic QLQ-C30 scores were also elevated, with higher results observed in the experimental group in contrast to the control group (*P* < 0.05). The research by Van Etten et al. [25] found that among 80 patients with brain metastases receiving WBRT, the patients given hippocampus sparing obtained better quality of life and better living abilities than those with hippocampus sparing therapy. Furthermore, the experimental group of this study obtained a superior prognosis of patients than the control group (*P* < 0.05), which suggests the inescapable impact of different brain metastases on patients' prognosis. The hippocampal volume of the control group was significantly smaller than that of the experimental group (*P* < 0.05), indicating that severe cognitive dysfunction is associated with small hippocampal volumes.

The application of hippocampal sparing in patients receiving whole-brain radiotherapy is effective in preventing cognitive dysfunction, improving the quality of life and prognosis of patients, and avoiding shrinkage of hippocampal volume. Encouragingly, Meduri et al. proposed that stereotactic radiosurgery be the treatment choice for patients with oligometastatic brain disease and a life expectancy of more than 3 months, and it should be considered an alternative to WBRT for patients with multiple brain metastases [26].

## Figures and Tables

**Table 1:** Technical methods of irradiation for both groups.

	Group with hippocampal sparing	Group without hippocampal sparing
Hippocampus	Contouring	Without contouring
Illumination area	Whole-brain radiotherapy+hippocampal sparing	Whole-brain radiotherapy
Illumination technique	Tomotherapy	IMRT
Illumination volume	2500 cGy/10f (PCI) or 3000 cGy/10f (WBRT)±local dosing	2500 cGy/10f (PCI) or 3000 cGy/10f (WBRT)±local dosing
CTV	Whole brain (fully including the dura)	Whole brain (fully including the dura)
PTV	0.5 cm CTV expansion	0.5 cm CTV expansion
Hippocampus PRV	0.5 cm expansion	None
Hippocampal PRV dose limitation	D40 < 7.3 Gy, Dmean < 10 Gy, Dmax < 17 Gy	None
OAR	Hippocampus, eye, lens, optic nerve	Eye, lens, optic nerve
OAR dose limitation	Dmax of eye < 25 Gy, Dmax of lens < 8 Gy, Dmax of optic nerve < 30 Gy	Dmax of eye < 25 Gy, Dmax of lens < 8 Gy, Dmax of optic nerve < 30 Gy

**Table 2:** Prognostic evaluation criteria for GPA grading.

Prognostic factors	Scores		
	0	0.5	1
Age	≥60	50~59	<50
KPS score	<70	70~80	90~100
Number of brain metastases	>3	2~3	1
Extracranial metastases	Yes	—	No

**Table 3:** Relationship between GPA grading and median survival.

GPA	Median survival (in months)
0~1	2.6
1.5~2.5	3.8
3	6.9
3.5~4	11

**Table 4:** Comparison of general information between the two groups (*n* (%)).

Items	Control group (*n* = 20)	Experimental group (*n* = 20)	*χ* ^2^/*t*	*P*
Gender			0.417	0.519
Male	11 (55.00)	13 (65.00)		
Female	9 (45.00)	7 (35.00)		
Average age (years)	58.37 ± 13.42	57.64 ± 12.93	0.175	0.862
GPA (score)	1.36 ± 0.11	1.39 ± 0.12	0.365	0.965
Education level				
High school and below	12 (60.00)	14 (70.00)	0.440	0.507
University and above	8 (40.00)	6 (30.00)		
Type of primary cancer				
Lung cancer	11 (55.00)	10 (50.00)	0.100	0.752
Breast cancer	5 (25.00)	7 (35.00)	0.476	0.490
Other	4 (20.00)	3 (15.00)	0.173	0.677

**Table 5:** Comparison of MoCA scores before and after radiotherapy between the two groups ($\overset{¯}{x}\pm s$).

Groups	Case	Before radiotherapy	3 months after radiotherapy	6 months after radiotherapy	12 months after radiotherapy
Control group	20	28.78 ± 1.63	26.71 ± 0.91	25.35 ± 2.04	24.19 ± 2.30
Experimental group	20	28.94 ± 1.55	27.46 ± 1.27	26.90 ± 2.15	26.11 ± 1.78
T		5.318	2.147	2.339	2.952
P		0.369	0.001	0.002	0.001

**Table 6:** Comparison of the clinical prognosis of patients between the two groups (*n* (%)).

Groups	Case	0~1	1.5~2.5	3	3.5~4
Control group	20	13 (65.00)	6 (30.0)	1 (5.00)	0 (0)
Experimental group	20	0 (0)	0 (0)	10 (50.00)	10 (50.00)
*X* ^2^					7.485
*P*					<0.05

## Data Availability

The datasets used and analysed during the current study are available from the corresponding author on reasonable request.
